# Abstracts from Foreign Journals

**Published:** 1902-06

**Authors:** S. J. J. Harger

**Affiliations:** Philadelphia, Pa.


### ABSTRACTS FROM FOREIGN JOURNALS.

### FRENCH.

UNDER THE DIRECTION OF S. J. J. HARGER, V.M.D.,
PHILADELPHIA, PA.

HERMAPHRODISM IN A BOVINE ANIMAL. The animal, about three and a half years old, had the general conformation of the castrated male, with a penis, two small teats, but no evidence of a scrotum nor the cicatrices of castration. He had the sexual ardor, and tried to mount other male or female members of the herd.

On opening the abdomen the following conditions existed: A perfectly developed female uterus, and attached to the anterior extremity of the broad ligament was a testicle on the right side and an ovary on the left, symmetrically situated. The testicle was softer than normal; the ovary was smaller than the testicle. Their histological structure was not as characteristic as the macroscopical appearance, and both resembled their common origin —the indifferent genital gland of the embryo.—*Sec. de Biologie.*

SUDDEN DEATHS OF GASTRIC ORIGIN. The vulgar diagnosis of "heart trouble," without any tangible evidence, is known to everyone. Lancereaux has made a necroscopical examination of fifty cases of this kind without finding any cardiac or aneurismal lesions. He believes that a common cause consists of nervous troubles of the stomach, and especially dyspepsia. Such fatal syncopes occur in consequence of the acute excitability of the heart by the troubles existing in the stomach. Sudden deaths are the result through reflex action of the instantaneous arrest of an essential function by altered nerve influences.—*Acad. de Méd.*

TRANSMISSIBILITY OF HUMAN TUBERCULOSIS TO CATTLE. Since 1890 Karlinski, of Bosnia (*Œstreich. Monat.*) inoculated twenty-five bovine animals, previously tuberculinized with the human tubercle bacillus, employing cultures on bouillon, and agar and fragments of tuberculous liver and lymph glands. In the ten animals which became infected the inoculations were made as follows: intrapleural, twice; intratracheal, intramammary, subcutaneous, testicular, each once; intraperitoneal, four times.

In fifteen cases the inoculations were negative, and the author concludes that the sensibility of the bovine organism to the bacillus of human origin is relatively limited. From one experiment it appears that the bacillus obtained from tuberculous lesions of a heifer inoculated with the human bacillus seemed to have increased its virulence, although positive proof of this requires further experimentation.—*Ann. de Méd. Vét.*

CHRYSOFORM. Chrysoform, a bromo-iodide of hexamethylenetetramin, is a yellowish powder emitting a slight odor of iodine. It is a powerful antiseptic and cicatrisant. It may be used as a powder, ointment, or emulsion in glycerin and water.

Cadiot especially recommends it in poll-evil, fistula of the withers, and the various quittors. In cartilaginous quittor the fistulous orifice is enlarged with a bistoury, and after arresting the hemorrhage the tract is filled with chrysoform and pushed as far toward its bottom as possible. This dressing is renewed every three or four days.

In fistula of the poll and withers the fistulous tract is washed out with an antiseptic solution, then packed with chrysoform, and its orifice tamponed with gauze.

Cadiot cites many observations in support of this treatment.—*Rec. de Méd. Vét.*

LUXATION OF THE CRYSTALLINE LENS. The adhesion between the crystalline lens and the zone of Zinn in the horse can be broken from violent compression of the ocular globe as well as by certain pathological states of the ciliary body. When displaced forward it falls into the anterior chamber of the eye, and often contracts adhesions with the membrane of Decemet, assuming a grayish-blue color. When dislocated backward into the vitreous humor it falls into the bottom of the posterior chamber, atrophies, and may become visible only with the ophthalmoscope.

Sometimes the luxation is incomplete, the lens becoming deviated at an angle. It soon becomes opaque, intercepting the rays of light, which causes the animal to hold his head to one side, in order to allow the light to pass through the free part of the pupil.

When the lens floats free in the anterior chamber it may be removed by incision of the cornea close to its border; when adherent to the cornea treatment is not recommended.—*Schimmel, in Ann. de Méd. Vét.*

Ligaturing of the Digital Artery in Founder. Joly relates three cases of ligaturing of the digital artery in acute and subacute osteitis of founder.

The first case was very aggravated, and the animal had to be relieved at once or destroyed. The external digital artery of both forefeet was ligatured, and the improvement was at once decided. The four hoofs were subsequently shed, but new hoofs of good shape were reproduced.

The second case, a thoroughbred horse, showed considerable deformity of the anterior right foot, accompanied by lameness. After the ligation of the artery the lameness disappeared, the deformed hoof was improved, and the animal was sent to work.

In a third case the results were similar.

The place of election for the ligaturing is the same as that for low plantar neurectomy. The artery is ligated with silk or catgut in two places and then sewed.

Joly recommends this treatment in all cases of acute or of subacute osteitis (laminitis) if the third phalanx has resisted the usual treatment.—*Rec. de Med. Vet.*

Avian Diphtheria. According to Guérin, chief of the laboratory institute of the Pasteur Institute of Lille, avian diphtheria is caused by a cocco bacillus, which is aërobic or anaërobic, mobile, taking Gram's stain, not liquefying gelatin nor coagulating milk whose reaction is not changed, not growing upon potatoes of natural acidity, not giving off indol. Old cultures have a special odor. The best culture medium consists of fresh peptonized bouillon, eight parts, and blood serum of the horse, one part.

Cultures obtained from chronic lesions in the chicken were sometimes pathogenic in the chicken, mouse, sparrow, pigeon, and rabbit. Their virulence has been very much increased. After fifteen passages through the pigeon, the inoculation being made into the lower eyelid, $\frac{1}{8}$ c.c. destroys the pigeon in fifteen to eighteen hours. With lesions of a rapid septicæmia, chickens offer much more resistance to inoculations than pigeons. The disease was also produced by cohabitation and by food and water contaminated with a virulent culture. The excrement is infectious, and from it the bacillus can be isolated. In some instances in the pigeon there was a scapulohumeral and humero-radial arthritis. The pigeon, being most susceptible, is best adapted for experimentation.

Preventive inoculations under the skin have not been successful. Guérin made the injection into the peritoneal cavity. The first injection is made with a virulent bouillon serum culture, twenty-four hours old, heated to 55° C., all the virulent agents being destroyed at this temperature ; $\frac{1}{2}$ c.c. is used. The second inoculation, made twelve days afterward, is similar to the first, but the culture is heated only to 50° C. ; the bacillus has not entirely lost its virulence. Twelve to fifteen days after the second injection, the animal will resist a fatal dose of a virulent culture. This procedure was applied practically with success in infected flocks. The chick to be so treated should be approaching its second month.

Guérin endeavored to produce an anti-diphtheritic serum. He immunized two horses by intravenous and intraperitoneal injections, following the usual procedures in the production of anti-microbic serums. One of these horses, treatment having commenced in April, furnished in November a serum of which 4 c.c. injected subcutaneously in the pigeon enabled the animal at the end of twenty-four hours to resist a fatal quantity, $\frac{1}{4}$ c.c. of a virulent culture injected intravenously ; 2 c.c. of the serum also resisted the same inoculation when made under the skin.

In serovaccination against diphtheria, as in swine plague, two inoculations are necessary at an interval of twenty-four hours ; the first, made subcutaneously, consists of a dose of the serum varying according to the susceptibility of the species to the disease ; the second, which is a virulent culture, is made in the manner indicated above.

The therapeutic use of the serum, which thus far has given little result, is under experimentation.—*Ann. de Med. Vet.*

Action of Water Drunk During Digestion. Leconté experimented upon this subject in the dog, and determined the following results :

1. After a meal composed of meat or bread, with or without potatoes, the water even drunk in abundance, passes principally along the smaller curvature of the stomach without at times entirely saturating the whole of the gastric contents. After fifteen minutes all the liquid passes out of the stomach, carrying with it only a little finely-powdered material held in suspension by the gastric secretion. Even in a case of extreme thirst only a few morsels of meat were found in the intestines fifteen minutes afterward.

In the intestine the water is absorbed as rapidly as it enters ; the organ is found in the same condition, whether or not the animal has received water, and the intestinal digestion is continued without any modification.

2. When the animal is fed on thin soup and then forced to drink water, the latter is added to the contents of the stomach, which becomes distended, producing malaise for a short time, and afterward surcharging the intestine.

The ingestion of liquids, according to the author, does not interfere with digestion in the dog, unless the quantity becomes large. In man, and this was the principal aim of the experiments, the results are most probably similar, and in sickness the thirst may be satisfied irrespective of the period of digestion. In gastro-intestinal affections the judicious use of water need not be withheld.—*Ann. de Med. Vet.*

Diagnosis of Foreign Bodies in the Œsophagus. The symptoms of obstruction of the œsophagus, such as loss of appetite, nausea, slobbering, tympanites, etc., are common to a number of other diseases, and may simulate a simple meteorization.

Zofir suggests the following simple procedure before using the bougie or making a manual examination of the pharynx: Give the animal about two litres of tepid water. In œsophageal obstruction the liquid will be ejected through the nostrils and the mouth. When the stenosis is incomplete or close to the stomach the stagnation of the liquid in the canal anterior to the obstruction will be noticeable.—*Rec. de Med. Vet.*

Corrosive Sublimate in Foot-and-Mouth Disease. Hirzel experimented upon seventy-three cows, twenty-five oxen, and ninety other head of cattle on a farm where the disease had existed two years before. Some of the animals therefore had acquired a certain degree of immunity, and the symptoms were benign. There was not the least improvement in the symptoms. As a preventive the intravenous injection of the bichloride was likewise without effect.

In spite of the minimum dose, the author has, in some cases, seen symptoms of mercurial poisoning, such as ptyalism and moist, serous, indurated patches, which, in the cow, were localized on the perineum, in the ox at the base of the tail, and in the calves around the mouth, head, and neck. In the young animals there were also muzzle, mouth, and nasal cavities which bled from the least friction. The nearby lymph was pulled off by the fingers, and the opening caused by this trouble was not found in animals not treated. The results seem to show that the bichloride treatment gives very little encouragement.—*Ann. de Med. Vet.*

